# Selected *N*-Terpenyl Organoselenium Compounds Possess Antimycotic Activity In Vitro and in a Mouse Model of Vulvovaginal Candidiasis

**DOI:** 10.3390/molecules28217377

**Published:** 2023-10-31

**Authors:** Xiuyi Liang, Agata J. Pacuła-Miszewska, Magdalena Obieziurska-Fabisiak, Richa Vartak, Ganming Mao, Ketankumar Patel, Natalya U. Fedosova, Jacek Ścianowski, Blase Billack

**Affiliations:** 1 Department of Pharmaceutical Sciences, St. John’s University, Queens, NY 11439, USA; xiuyi.liang18@my.stjohns.edu (X.L.); richa.vartak17@my.stjohns.edu (R.V.); ganming.mao18@my.stjohns.edu (G.M.); patelk2@stjohns.edu (K.P.); 2 Faculty of Chemistry, Nicolaus Copernicus University, 87-100 Toruń, Poland; pacula@umk.pl (A.J.P.-M.); magdao@umk.pl (M.O.-F.); jsch@chem.umk.pl (J.Ś.); 3 Department of Biomedicine, Aarhus University, 8000 Aarhus, Denmark; nf@biomed.au.dk

**Keywords:** *Candida albicans*, vulvovaginal candidiasis, *N*-terpenyl organoselenium, antifungal activity, *Candida* resistant strains

## Abstract

In the present work, a series of *N*-terpenyl organoselenium compounds (CHB1-6) were evaluated for antimycotic activity by determining the minimum inhibitory concentration (MIC) for each compound in fluconazole (FLU)-sensitive (S1) and FLU-resistant (S2) strains of *Candida albicans* (*C. albicans*). The most active compounds in the MIC screen were CHB4 and CHB6, which were then evaluated for cytotoxicity in human cervical cancer cells (KB-3-1) and found to be selective for fungi. Next, CHB4 and CHB6 were investigated for skin irritation using a reconstructed 3D human epidermis and both compounds were considered safe to the epidermis. Using a mouse model of vulvovaginal candidiasis (VVC), CHB4 and CHB6 both exhibited antimycotic efficacy by reducing yeast colonization of the vaginal tract, alleviating injury to the vaginal mucosa, and decreasing the abundance of myeloperoxidase (MPO) expression in the tissue, indicating a reduced inflammatory response. In conclusion, CHB4 and CHB6 demonstrate antifungal activity in vitro and in the mouse model of VVC and represent two new promising antifungal agents.

## 1. Introduction

Vulvovaginal candidiasis (VVC), a disease characterized by acute inflammation of the vulval and vaginal mucosa, impacts millions of women annually, resulting in significant economic costs both directly and indirectly [1,2]. VVC is characterized by symptoms including vaginal discharge, pruritus, and dyspareunia [1,3]. Typically, VVC is caused by the overgrowth of *Candida albicans* (*C. albicans*) in the vaginal tract [4] and has been found to recur in up to 8% of women globally [4,5,6]. This interesting microbe can exist in distinct forms and switch phenotype from the usually harmless yeast form to the infectious hyphal form [4,7] and these different morphologies play a significant role in its virulence [6,7,8].

The primary objective in treating VVC is to stop the overgrowth of yeast in the vaginal tract. Clinical isolates of *C. albicans* obtained from patients with VVC and exhibiting resistance to the standard antifungal drug fluconazole (FLU) have been observed; moreover, resistance to FLU appears to be more common in VVC than previously believed [9,10]. Indeed, the emergence of resistance to FLU and other antifungal drugs undermines the effectiveness of currently marketed antifungal medications and increases the need for new medicines with efficacy towards *Candida* spp. [11,12,13]. Therefore, novel antifungal drugs are needed.

Ebselen (EB) is a synthetic organoselenium compound that has been extensively studied in numerous in vitro and in vivo research settings [14,15,16]. Interestingly, EB has demonstrated effectiveness as an inhibitor of the plasma membrane H^+^-ATPase proton pump (Pma1p), which plays a critical role in fungal survival [17,18,19]. Due to its distinctive bioactivity, EB has shown promise as a valuable agent in the treatment of VVC, offering potential therapeutic benefits for this condition [20]. However, the potential toxicity of EB and its propensity for off-target interactions with sulfhydryl-containing enzymes remains a concern [21,22].

**Figure 1:**
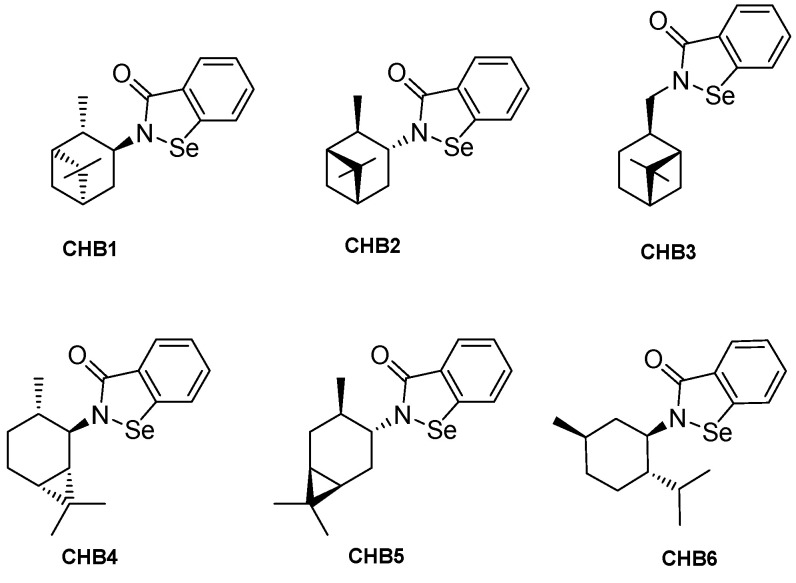
Chemical structures of the *N*-terpenyl organoselenium compounds tested here.

In an effort to develop organoselenium compounds that retain the potential therapeutic benefits of EB while reducing the potential for toxicity, six novel N-substituted terpenyl organoselenium compounds, namely CHB1-6, were previously synthesized and found to possess antioxidant and anti-proliferative activities [23]. In the present work, we have evaluated the antifungal activities of these novel *N*-terpenyl analogs of EB and compared them to EB and to the commercially available antifungals FLU and miconazole (MICO) in clinically relevant strains of *C. albicans*. To assess the antimycotic activity of these CHB compounds (Figure 1), two approaches were utilized:

(i) in vitro growth inhibition and medium acidification experiments, and
(ii) an in vivo experiment using a mouse model of VVC.

Taken together, the experiments presented below demonstrate that selected CHB compounds, namely CHB4 and CHB6, are highly effective towards *C. albicans*. Lastly, an evaluation for acute toxicity demonstrated the safety of the two most active analogs.

## 2. Results

### 2.1. Chemical Properties and MIC Determination

Prior to beginning our work, high-performance liquid chromatography (HPLC) analysis was performed to evaluate the purity of the six CHB test compounds. The chemical properties of the test compounds were predicted by the SwissADME platform and are shown in Table 1. The predicted water solubilities of the test compounds ranged from the lowest value of 2.75 µg/mL (CHB6) to the highest value of 6.30 µg/mL (CHB5). All the test compounds showed a lower predicted water solubility than that predicted for EB (33 µg/mL). The HPLC results showed high purity of test compounds (Supplementary Materials Figures S1 and S2).

We next determined the minimum inhibitory concentration (MIC) of the test compounds in turbidity assays. As shown in Table 2, the MIC of EB-treated wells in both S1 (FLU-sensitive) and S2 (FLU-resistant) strains was 25 µM after 24 h and 48 h incubation. In the S1 strain, CHB1, CHB2, and CHB4 showed similar MIC values of 12.5 µM after both 24 h and 48 h while CHB5 showed a MIC of 12.5 µM after 48 h only. CHB3 was less potent than the others, showing MIC values of 25 µM at both time points, which was similar to the EB-treated wells. On the other hand, CHB6 exhibited strong potency with MIC values of 3.1 µM after both 24 h and 48 h in both S1 and S2 strains. Interestingly, CHB1, CHB4 and CHB6 were more potent than EB, CHB2, CHB3, and CHB5 in the S2 strain at either 24 or 48 h. Because FLU is a fungistatic compound, the MIC for FLU was regarded as the lowest concentration of FLU causing a 70% decrease in turbidity compared to the growth of control wells. To this end, the MIC for FLU in the S1 strain was found to be 25 µM at both 24 and 48 h while this value was > 100 µM in the S2 strain confirming its FLU resistant phenotype.

To affirm the turbidity studies, we determined the MIC values of the test compounds using a colorimetric resazurin assay (Table 3). The resazurin dye is often used as an indicator of living cells [24]. In the S1 strain, the MIC values of CHB6-treated wells were 3.1 µM after either 24 h or 48 h incubation, whereas the MIC of CHB4 was 6.3 µM after 24 h but 12.5 µM after 48 h. The other compounds (CHB1, CHB2, CHB3, CHB5 and EB) were less potent in the S1 strain compared to either CHB4 or CHB6. A representative image of colorimetric assays in the S1 strain is shown in Supplementary Materials Figure S3. The EB-treated wells in both S1 and S2 exhibited MIC values of 25 µM after 24 h or 48 h incubation. In the S2 strain, CHB4 was the most potent at both 24 h and 48 h (MIC = 3.1 µM at both time points), followed by CHB2 (MIC_24h_ = 6.3 µM; MIC_48h_ = 3.1 µM) and CHB6 (MIC_24h_ = 6.3 µM; MIC_48h_ = 6.3 µM).

### 2.2. Comparing Half Maximal Inhibitory Concentrations (IC_50_ Values) in KB-3-1 Cells and C. albicans

The determination of IC_50_ in *C. albicans* (Table 4) was derived from turbidity assays by measuring the turbidity of treated samples spectrophotometrically at Abs_600nm_ following the MIC evaluations at 48 h. In the S1 strain, CHB6 exhibited the highest potency with an IC_50_ of 1.8 µM. Compounds CHB1, CHB2, CHB4 and CHB5 showed IC_50_ values in the range of 7.3 to 7.7 µM while CHB3 showed an IC_50_ value of 18.1 µM, which was like the IC_50_ for EB (~17 µM). In the S2 strain, again, CHB6 was the most potent (IC_50_ = 1.8 µM). Interestingly, CHB1 and CHB4 were equipotent to CHB6 in this strain. Compounds CHB2, CHB3 and CHB5 showed IC_50_ values in the range of 6.2 to 15.1 µM, which was like the IC_50_ for EB in the S2 strain (~14 µM). The IC_50_ for FLU in S1 was ~6 µM, while in the S2 strain the IC_50_ was >100 µM (the highest test concentration), affirming that S1 was FLU-sensitive while S2 was FLU-resistant.

Overall, the most potent CHB analogs in both the MIC assays and IC_50_ analyses were CHB4 and CHB6.

MTT assays were then performed to assess the effect of the test compounds on human epidermoid carcinoma cells KB-3-1 (24 h incubation). The results are shown in Table 4. CHB1 and CHB2 showed antiproliferative activity in KB-3-1 with the IC_50_ of 28.1 µM and 25.5 µM, respectively. CHB3 displayed antiproliferative activity at >60 µM. CHB4 exhibited the most potent antiproliferative activity in KB-3-1 cells among these compounds with the IC_50_ of 17.9 µM in comparison to EB (IC_50_ = 89.7 µM). CHB6 exhibited a less potent antiproliferative activity in KB-3-1 cells with the IC_50_ of 48.5 µM. The IC_50_ for CHB5 was >90 µM while for FLU-treated cells the IC_50_ > 1000 µM, revealing no antiproliferative activity of either CHB5 or FLU in KB-3-1 cells. It should be noted that small crystals of CHB5 were observed under the microscope in KB-3-1 cells treated with CHB5 concentrations above 50 µM, thus, it was not considered further here. Moreover, the HPLC trace of CHB5 (Supplemental Figure S2, Panel B) revealed a small second peak, suggesting it may be unstable or may have contained a minor impurity.

Overall, the fungal selectivity for the CHB test compounds (derived from the ratio of the IC_50_ in KB-3-1 cells to the IC_50_ in S1 cells) ranged from 2.3 for CHB4 to 26.9 for CHB6, indicating that the CHB test compounds were more selective for the S1 yeast than for the human cervical cells.

### 2.3. Effect on Medium Acidification in C. albicans S1

Typically, yeast cells export protons from their intracellular compartment to the extracellular environment using a pump known as the yeast plasma membrane H^+^-ATPase (Pma1p). The inhibition of Pma1p prevents protons from entering the extracellular medium, keeping the pH of the external medium stable [25]. Here, a medium acidification assay was utilized to detect the effects of the CHB test compounds on Pma1p in the S1 strain. Of all the CHB test compounds, only CHB4 exhibited concentration-dependent inhibition of medium acidification with an IC_50_ (IC_50MA_) of ~14 µM (Table 5). The other compounds, CHB1, CHB2, CHB3, CHB5, CHB6 and FLU did not inhibit medium acidification at test concentrations up to 30 µM (Table 5). EB inhibited medium acidification in the S1 strain in a concentration-dependent way (IC_50MA_ = 12.5 µM) (Table 5). It is noteworthy that EB, CHB4 and CHB6 were tested and found to possess inhibitory activity towards the Na^+^, K^+^-ATPase (purified from pig kidney outer medulla) at test concentrations similar to the MIC concentrations described above (see Supplementary Materials, Figure S4).

### 2.4. Efficacy of Treatments in the Mouse Model of VVC

**Figure 2:**
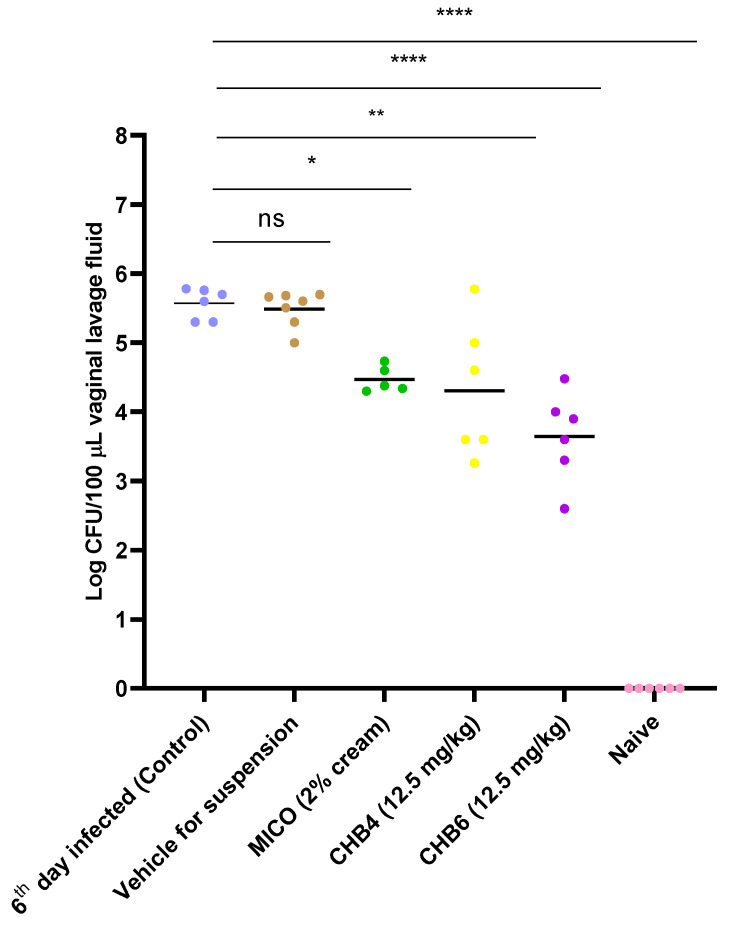
Efficacy of CHB4 and CHB6 (12.5 mg/kg) in the mouse model of VVC. Data shown are the CFUs obtained from each animal from the respective groups. The fungal burden of murine VVC was calculated on a scale of log10 (CFU/100 µL) in the vaginal lavage. MICO served as a positive control group for treatment. Points on the curve represent individual mice while horizontal lines represent the geometric mean of each group. * *p* < 0.05 vs. 6th day infected, ** *p* < 0.01, **** *p* < 0.0001 vs. 6th day infected.

CHB4 and CHB6 were selected for an intervention study in the mouse model of VVC carried out using *C. albicans* S1 (Supplementary Materials, Figure S5). On days 3, 4, and 5, mice with VVC received respective treatments via the intravaginal route once per day. On day 6 post-inoculation, mice were euthanized and vaginal lavage fluid was collected, and the measurement of colony forming units (CFU/100 µL) was performed on YPD agar plates. The vaginal fungal burden indicated that the 6th day infected group showed a high infection rate. Treatment with CHB4 suspension reduced the fungal burden with only ~7% of yeast remaining in the lavage, which is as potent as MICO in the reduction of *C. albicans* S1 (Table 6, Figure 2). The CHB6 treatment group showed ~1% of the CFU compared to control group. It is noteworthy that the vehicle group showed a similar percentage of remaining yeasts in the lavage compared to the control group (~100%). This indicated that the vehicle containing DMA and 1% HPMC had no major activity towards the yeast.

### 2.5. Histological Analysis of the Vaginal Tissue

**Figure 3:**
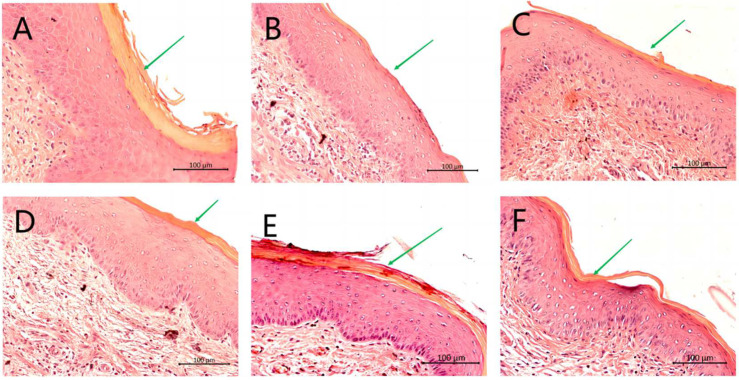
Histopathological analysis of vaginal tissues with H&E staining. Mouse vaginal tissue was excised longitudinally. Each section of paraffin-embedded tissues was stained with H&E and then observed using light microscope. (**A**) Naive; (**B**) 6th day infected; (**C**) vehicle; (**D**) MICO; (**E**) CHB4; (**F**) CHB6. Magnification: 200×; scale bars: 100 μm.

Vaginal tissues of mice were excised longitudinally, fixed in formalin and stained with H&E. Tissue sections from Naive group (uninfected, untreated) (Figure 3, Panel A) showed no epithelial hyperplasia with the green arrow pointing to a well-defined keratin layer. Tissues from 6th day group (infected, untreated) show epithelial hyperplasia and the green arrow demonstrates that the keratin layer is thin or missing (Figure 3, Panel B). The vehicle group showed edema and hyperplasia of the vaginal epithelial cells, with the green arrow pointing to a thin but well-defined keratin layer (Figure 3, Panel C). The mice treated with MICO, CHB4 or CHB6 (Figure 3, Panels D, E and F, respectively) showed slight epithelial distress with the green arrows pointing to stratified keratinized epithelium, indicating the effective treatment of VVC by these agents. No histopathological signs of toxicity were observed in the acute toxicity study where mice were intravaginally exposed to these test compounds in the absence of a yeast infection (Supplementary Materials, Figure S6).

### 2.6. Immunohistochemical Analysis

Myeloperoxidase (MPO) is an enzyme which is present in the polymorphonuclear neutrophils (PMNs) that provide the first line of defense to the host in the inflammatory responses [26].

**Figure 4:**
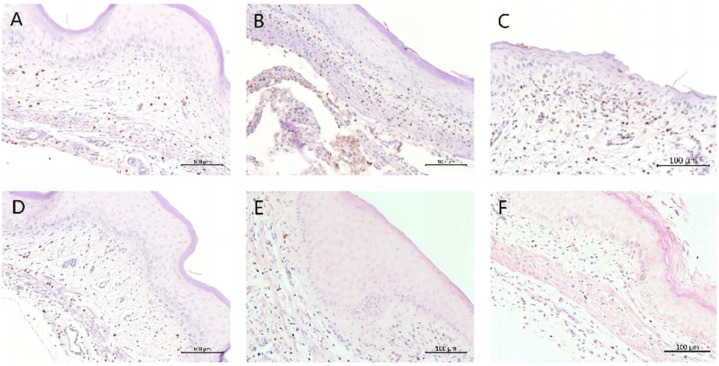
Immunohistochemistry analysis of vaginal tissues with an antibody to MPO. (**A**) Naive; (**B**) 6th day infected; (**C**) vehicle; (**D**) MICO; (**E**) CHB4; (**F**) CHB6. Magnification: 200×; scale bars: 100 μm.

IHC analysis of vaginal tissues showed a slight presence of MPO in the Naive group (Figure 4, Panel A). In the 6th day infected group, high levels of MPO were observed (Figure 4, Panel B), demonstrating the inflammatory response during VVC infection. The CHB4- and CHB6-treated groups showed a decrease (Figure 4, Panels E and F, respectively) in expression of MPO that was similar in MPO expression as that of the MICO treated group (Figure 4, Panel D). Thus, compared to the 6th day infected and vehicle groups (Figure 4, Panel C), the lower expression of MPO observed in the CHB4- and CHB6-treated groups indicated the reduced inflammatory response due to the treatment.

**Figure 5:**
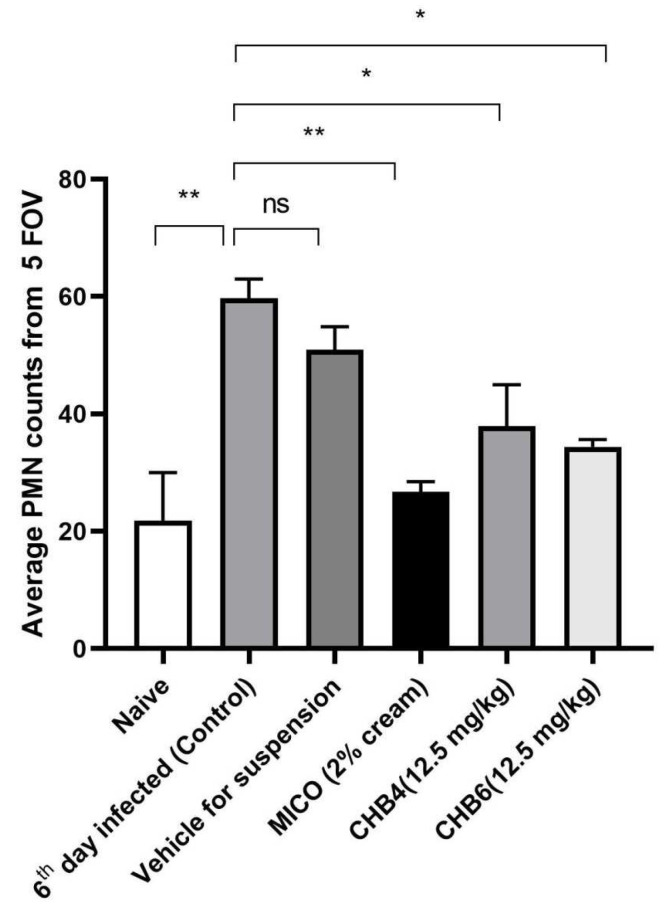
PMN counts of vaginal tissues. Sixth (6th) day infected group showed high amount of PMN. CHB4 and CHB6 groups showed less PMN counts compared to control and vehicle group. Each bar represents the average of three tissues per treatment. Data are expressed as mean ± SEM (n = 4). * *p* < 0.05 or ** *p* < 0.005.

The number of MPO cells in five fields of view (FOV) for each section were then counted and the average was plotted on a graph (Figure 5). To summarize, both CHB4 and CHB6 caused a dramatically reduced inflammatory response with fewer PMNs presence and an antifungal efficacy comparable to MICO in the mouse model of VVC (Figure 5).

### 2.7. In Vitro Study on Reconstructed Human Epidermal Model

**Figure 6:**
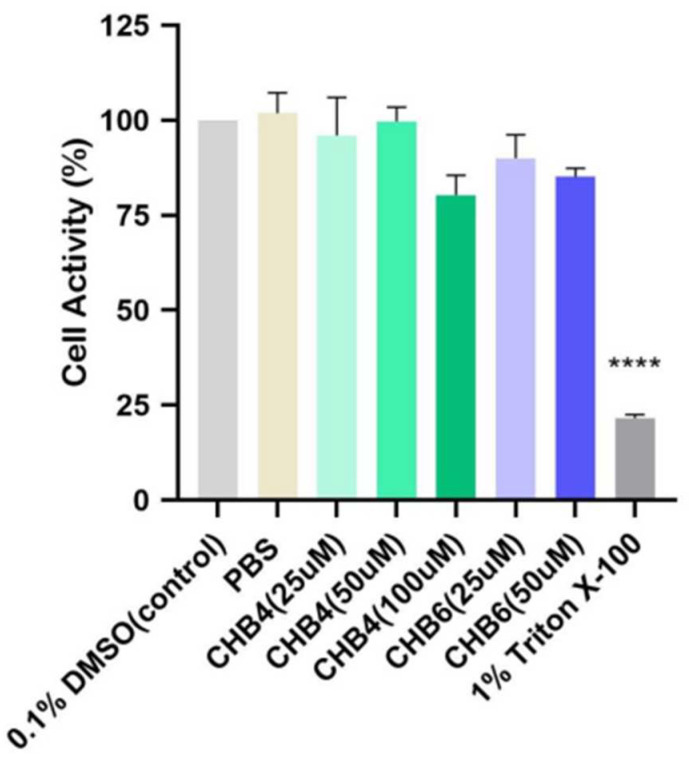
Skin irritation test of CHB4 and CHB6 on reconstructed human epidermal model. The data were analyzed by one-way ANOVA. **** *p* < 0.0001.

Using reconstructed human epidermis (Mattek Epiderm) and OECD test guideline No. 439 [27], no skin irritation was observed at test concentrations of CHB4 up to 100 µM or CHB6 up to 50 µM (Figure 6).

## 3. Discussion

VVC is characterized by an acute inflammatory infection that primarily affects women in their reproductive years. It has implications for their social and work life, leading to emotional and psychological distress due to feelings of embarrassment and the presence of societal stigma in certain cultures [28]. Although azoles are commonly used as the standard antifungal treatment for VVC and are easily accessible, their effectiveness is limited, and there has been an increase in antifungal resistance. Additionally, azoles are fungistatic, meaning they inhibit the growth of fungi rather than killing them outright, which contributes to the recurrence and persistence of fungal infections. Orally administered FLU has been widely prescribed over the past two decades for the treatment of VVC. However, the extensive use of FLU has resulted in the emergence of FLU-resistant strains of *Candida*, posing a significant obstacle to the successful treatment of VVC [10,29].

EB, the prototype organoselenium, has been shown by our lab and others to exert antimycotic effects [17,18,19,20,21,30,31]. Moreover, EB administered by the intravaginal route serves a dual purpose: (i) it effectively addresses VVC at the specific location, and (ii) it serves as a pre-exposure prophylaxis against HIV [32]. However, while EB shows promise as an effective antimycotic agent, it possesses several limitations in practical work, such as low water solubility and broad pharmacological effects. To potentially circumvent such limitations, new *N*-terpenyl organoselenium compounds, namely CHB1-6, have recently been synthesized [23]. To investigate their ability to inhibit yeast cell growth, we conducted colorimetric and turbidity assays to determine the MIC for each test compound (Table 2 and Table 3).

The highest antimicrobial activity was observed for (−)-N-(1R,2S,5R)-menthyl-1,2-benzizoselenazol-3(2H)-one (CHB6). This result corresponds with our previous analysis concerning the cytotoxic potential of N-terpene benzisoselenazolones. In particular, the anticancer activity towards breast cancer cell line MCF-7 was also most efficiently expressed by compound CHB6 with an observed IC_50_ of 11.9 ± 0.2 µM [23]. We assume that the 3-methylbutyl carbon chain, incorporated in the structure of the N-menthyl derivative CHB6, can act as a pharmacophore responsible for the drug-target interaction. The same structural motif was also present in the structure of other active benzisoselenazolones–N-3-methylbutyl and N-leucine analogues [33,34]. The lowest antimicrobial capacity observed for the (−)-N-(1S,2R,5S)-myrtanyl-1,2-benzisoselenazol-3(2H)-one CHB3 can be explained by the distance of the chiral terpenyl skeleton from the bioactive Se-center which is elongated by an additional -CH2- bridge. In the case of the N-isopinocampheyl enantiomers, CHB1 and CHB2, and the N-caranyl regioisomers CHB4 and CHB5, the similar potency observed here may be due to the bulky bicyclic system that forms the core of the chiral scaffold.

The plasma membrane H^+^-ATPase (Pma1p) plays a crucial role in nutrient uptake and stress response in fungal physiology [35]. Due to its regulation by various nutritional and environmental factors, Pma1p is an attractive target for antifungal agents [36,37]. We conducted medium acidification assays using the *N*-terpenyl organoselenium compounds and observed their effects on Pma1p (Table 5). Among the test compounds, only EB and CHB4 showed concentration-dependent suppression of acidification effects in the *C. albicans* S1 strain. The reason why only CHB4 inhibited this pump remains unclear. The substituent group carane in CHB4 is more likely to hinder the Se-N bond, which may improve the drug target interaction with Pma1p. It should be noted that the medium acidification assay, which progresses over a period of 30 min, is performed only after a preliminary incubation of 2 min using intact yeast cells and may require a longer pre-incubation step when used in different analogs of EB. Moreover, the possibility remains that the antifungal activity of CHB6, found to be the most potent test compound in vitro, may be inhibiting yeast growth through a mechanism that does not involve Pma1p inhibition. It may also be that the inhibition of Pma1p by EB or CHB4 is unrelated to their antimycotic effects. These are all points that require clarification and are worthy of future study.

The CHB4 and CHB6 compounds were found to inhibit the Na^+^, K^+^-ATPase, an enzyme critical for animal cells (host) homeostasis. The inhibiting effect observed in the micromolar range (Supplementary Materials, Figure S4) was practically irreversible but prevented by the presence of GSH in physiological intracellular concentrations. Thus, the reactivity towards off-target sulfhydryl-containing enzymes poses a significant obstacle for the systemic administration of the EB or the CHB4 or CHB6 analogs. However, when CHB4 or CHB6 were administered intravaginally as a suspension (present experiments), they each exerted significant antifungal activity without affecting any gross markers of the health of the mice (e.g., the mice remained responsive and active and did not lose weight over the course of the study). Thus, through the intravaginal route of administration, the use of organoselenium compounds such as CHB4 or CHB6 to treat VVC remains plausible.

It should be noted that the clinical isolates S1 and S2 utilized in this study were originally obtained from an AIDS patient who experienced oral mucocutaneous candidiasis [38]. These two strains were selected to demonstrate the potent efficacy of EB and the CHB test compounds in both FLU-sensitive and FLU-resistant strains in vitro. However, only the S1 strain was employed to establish the vaginal candidiasis (VVC) infection in the mouse model. It should be acknowledged that the fact that the S1 strain originates from the oral cavity rather than the vaginal cavity is a limitation of our research. Nonetheless, we found that S1 exhibited excellent colonization ability, likely due to its origin from a mucocutaneous oral environment. It is important to note that the vaginal tract is also a mucocutaneous environment. However, the rodent vagina is close to pH neutral and is not colonized by *Lactobacillus* spp., which are two aspects that were not considered in the present work [39,40]. Thus, future investigation of CHB4 or CHB6 as a treatment for VVC in humans will be needed to determine the extent to which the human vaginal pH and microbiome impact the antimycotic efficacy of these novel organoseleniums.

Interestingly, we used YPD containing dextrose for the yeast inoculation step. However, the optimal carrier for yeast inoculation is PBS or synthetic vaginal fluid. Thus, our use of YPD may help to explain the mouse-to-mouse variability observed in fungal colonization across control and treatment groups (see Figure 2). And while YPD is not the preferred carrier fluid for inoculation, we have previously used it and observed vast numbers of blastoconidia and filamentous pseudohyphae adhering to the vaginal epithelium 6 days post-inoculation with yeast [31]. In the future, however, our group will utilize PBS or synthetic vaginal fluid as the carrier fluid during the yeast inoculation step to ensure harmonization within the mouse model of VVC. In addition, the experimental design used here for the mouse VVC model (Supplemental Figure S5) wherein vaginal lavage fluid and vaginal tissues are and analyzed 6 days after inoculation is like Garvey and colleagues who assessed fungal burden in vaginal lavage fluid from infected mice 7 and 10 days after yeast inoculation [41].

After having identified the most active compounds using the in vitro screens described above, we performed an in vivo study using the mouse model of VVC, specifically focusing on CHB4 and CHB6. After 3 days of intravaginal dosing (once per day, 12.5 mg/kg), the treated groups exhibited a significant reduction in fungal burden. Only 6.607% (CHB4) and 1.064% (CHB6) of yeast remained in the lavage when compared to infected, untreated mice (Figure 2, Table 6). Histological analysis using H&E staining revealed that mice infected with yeast but treated with CHB4 or CHB6 exhibited a vaginal epithelium more closely resembling vaginal tissue obtained from naïve, uninfected mice. Additionally, immunohistochemical analysis demonstrated reduced expression of MPO, indicating a significant decrease in the inflammatory response and a lower presence of PMNs.

It should be emphasized that only the S1 strain was used here to establish VVC infection in the mouse model. Future work will be required to determine the extent to which CHB4 and CHB6 inhibit VVC infection by FLU-resistant strains of *C. albicans* such as S2 and others. It is also not clear at the present time whether these compounds exhibit broad antifungal utility in other FLU-resistant non-albicans species of *Candida* or in other clinically relevant fungi (e.g., *Cryptococcus neoformans*). Thus, future studies will be required to determine whether the *N*-terpenyl organoseleniums exert broad or narrow antifungal effects.

Organoselenium compounds generally exhibit poor water solubility and high reactivity with both protein and nonprotein thiols. During the review of this manuscript, it was pointed out that nanoparticles prepared from inorganic selenium might be able to avert some of the limitations of organoselenium compounds and allow for selective targeting of yeast while avoiding non-selective effects in the host. The use of selenium nanoparticles to treat VVC is a particularly intriguing idea, as several recent studies have attributed antimicrobial and antiviral effects to these nanoparticles [42,43,44]. While beyond the scope of the present work, a future study in this area is warranted.

### 3.1. Conclusions

Organoselenium compounds are showing promise as antifungal candidates. The present study describes the antimycotic activity of two novel organoselenium compounds, CHB4 and CHB6, and shows that these compounds inhibit *C. albicans* growth in a manner that is more potent than the prototype organoselenium EB. Whereas these two CHB compounds also exert similar effects in the S2 fluconazole-resistant strain, in vitro studies demonstrate that they do not affect the viability of KB-3-1 cells at test concentrations that inhibit fungal growth, indicating a preference for fungal cells. Furthermore, like the standard intravaginal drug MICO, both CHB4 and CHB6 reduce VVC in a mouse model. Overall, the work here has identified two new promising drug candidates for VVC treatment.

### 3.2. Summary Points

- Vulvovaginal candidiasis (VVC) is often referred to as, “vaginal yeast infection”.
- Two potent and novel *N*-terpenyl analogs of ebselen (EB), named CHB4 and CHB6, were characterized for anti-*Candida* efficacy.
- CHB4 and CHB6 inhibited the growth of two clinical isolates of *C. albicans* in vitro.
- CHB4 inhibited the plasma membrane H^+^-ATPase (Pma1p), an essential protein in *C. albicans*, indicating that this mechanism is involved, at least in part, in its antifungal activity.
- EB and the *N*-terpenyl analogs CHB4 and CHB6 all inhibited the Na^+^, K^+^-ATPase, indicating that this class of compounds has the potential to exhibit off-target effects.
- CHB4 and CHB6 were not irritating to reconstructed human epidermis.
- CHB4 and CHB6, administered intravaginally, both showed potent efficacy in the mouse model of VVC.
- Histopathologic analysis of vaginal tissues from CHB4 or CHB6-treated mice showed less inflammation and tissue injury than tissues obtained from infected but untreated control mice.
- CHB4 and CHB6 represent new *N*-terpenyl analogs of EB that should be investigated as potential treatments for human VVC.

## 4. Materials and Methods

### 4.1. Chemicals and Strains

The strains of *C. albicans* used in this study were either sensitive to FLU (clinical isolate S1) or resistant to FLU (clinical isolate S2) and were provided by Dr. J. Morschhäuser (University of Würzburg, Würzburg, Germany). The KB-3-1 cell line was kindly provided by Dr. Zhe-Sheng Chen (St. John’s University, Jamaica, NY, USA). Balb/c females at 6 weeks of age were purchased from Taconic Laboratories, Inc. (Albany, NY, USA).

The synthesis of the CHB compounds has been previously described [23]. EB was purchased from AK Scientific, Inc. (Union City, CA, USA). FLU was purchased from ApexBio (Boston, MA, USA). DMA, estradiol valerate, RPMI 1640 media, DMSO and Tween 80 were acquired from Sigma Aldrich (St. Louis, MO, USA). Yeast-extract-peptone-dextrose (YPD) liquid and agar media were purchased from BD Diagnostic Systems (MD, USA). PBS and DMEM (Dulbecco’s Modified Eagle’s Medium) were purchased from Corning (Gilbert, AZ, USA). The MTT Reagent was purchased from Spectrum Chemical Mfg. Corp. (Gardena, CA, USA). Myeloperoxidase antibodies and the antigen retrieval solution were provided by Abcam (Waltham, MA, USA). MICO cream (2%) was purchased from a local pharmacy (PA, USA). Reconstructed human epidermal model EpiDerm™ was purchased from MatTek Corporation’s (Ashland, MA, USA). ABC reagent and DAB were purchased from Vector Labs (Newark, CA, USA). TBS was provided by Quality Biological Inc. (Gaithersburg, MD, USA).

### 4.2. Determination of Minimum Inhibitory Concentration (MIC)

For in vitro studies, DMSO was used to dissolve the six terpenyl compounds CHB1-6, FLU, and EB. The *C. albicans* strains S1 and S2 were cultured in YPD medium overnight before the turbidity assay. The *C. albicans* suspension was adjusted to achieve an initial inoculum with an Abs_600nm_ of 0.010 in RPMI 1640 medium. Serial dilutions of the test compounds were prepared by a 1:2 dilution ratio in RPMI 1640 medium. Each well of a 24-well plate was filled with 200 μL of the yeast suspension followed by 200 μL of the appropriate test compound solutions, resulting in concentrations ranging from 100 μM to 0.39 μM. FLU served as a positive control. The plates were incubated at 30 °C for 24 or 48 h and MIC values were recorded. The MIC was determined as the lowest concentration that completely inhibited the growth of *C. albicans*. The Abs_600nm_ values were also evaluated by spectrophotometer (Shimadzu Corp., Kyoto, Japan) to determine the IC_50_ value of these compounds.

For the colorimetric assay, all the test compounds were tested in both S1 and S2 strains of *C. albicans*. The yeast suspension was adjusted to achieve an initial inoculum with an Abs_600nm_ of 0.010 in RPMI 1640 medium. In 96-well plates, 100 μL of the yeast suspension was combined with 100 μL of the appropriate test compound solutions, resulting in concentrations ranging from 100 μM to 0.39 μM. The plates were incubated at 30 °C. After 24 h or 48 h incubation, 20 μL of 0.02% *w*/*v* resazurin dye was added to each well, and the change in dye color was observed to determine MIC values.

### 4.3. Evaluation of Medium Acidification Effects

A medium acidification assay was conducted to assess the inhibitory effects of the test compounds on the acidification of the growth medium caused by *C. albicans* S1 [11,18]. FLU was used as a negative control and EB served as the positive control in these assays. The IC_50MA_ was determined from a plot of the change in pH at 30 min versus the concentration of the test compound and was compared with the results for untreated cells.

### 4.4. Cell Viability Assay

The cell viability assay was performed in human epidermoid carcinoma cells KB-3-1. Initially, 5 × 10^3^ cells were seeded into each well in a 96-well plate. The following day, the cells were exposed to various concentrations of the test compounds or the control FLU. After 48 h of treatment, 20 µL of MTT solution (5 mg/mL) was added to each well and allowed to incubate for 3.5 h. Subsequently, DMSO was added to each well upon removal of the MTT solution. The Abs_570nm_ values were then measured using an accuSkanTM GO UV/VIS Microplate Spectrophotometer (Fisher Sci., Fair Lawn, NJ, USA).

### 4.5. Antifungal Activity in the Mouse Model of VVC

The mouse model utilized in this study serves as a valuable tool for investigating *Candida* infection pathogenesis and evaluating potential antifungal therapies in vivo [45]. Female BALB/c mice (6 weeks) weighing 18–-22 g were housed in an Association for Assessment and Accreditation of Laboratory Animal Care (AAALAC)-approved Animal Care Centre (ACC) at St. John’s University (Jamaica, NY, USA). The study was conducted following the guidelines and approval of the Institutional Animal Care and Use Committee (IACUC) of St. John’s University (Protocol #2003). The murine VVC study accounts for a total of 10 days, as shown in Supplementary Materials, Figure S5. Treatment groups contained 5–7 mice per group. We injected β-estradiol valerate, at a dose of 0.2 mg dissolved in 100 µL sesame oil subcutaneously on day −3 and day 3. This step aimed to place the mice in pseudoestrus so that yeast infection of the vaginal tract would be robust [46]. To this end, 20 µL of YPD medium containing *C. albicans* S1 (5 × 10^5^ CFU) was administered in the vagina on day 0, allowing the infection to progress until day 6. After euthanization on day 6, tissues were collected and fixed for H&E staining, as described previously [11,31]. On days 3, 4, and 5, the animals received the following treatments intravaginally:

- Group 1: Naive: non-infected (no treatment);
- Group 2: 6th day infected: estrogenized infected (inoculated with yeast) with no additional treatments (baseline value for infection);
- Group 3: MICO (2% cream): estrogenized infected (inoculated with yeast) treated with 27 µL of 2% MICO cream (positive control);
- Group 4: Vehicle: estrogenized infected (inoculated with yeast) treated with a 27 µL mixture of DMA (2.7 µL) + 1% HPMC (24.3 µL);
- Group 5: CHB4 (12.5 mg/kg): estrogenized infected (inoculated with yeast) treated with 27 µL suspension of 0.25 mg CHB4 dissolved in DMA (2.7 µL) + 1% HPMC (24.3 µL).
- Group 6: CHB6 (12.5 mg/kg): estrogenized infected (inoculated with yeast) treated with 27 µL suspension of 0.25 mg CHB6 dissolved in DMA (2.7 µL) + 1% HPMC (24.3 µL).

### 4.6. Immunohistochemistry

Vaginal tissue sections, measuring 5 μm in thickness, were obtained from formalin-fixed, paraffin-embedded samples. After hydration, the paraffin-embedded vaginal tissues were subjected to overnight incubation with an antigen retrieval solution. To elaborate, the tissues were treated with a primary antibody against myeloperoxidase (Abcam, Waltham, MA, USA; ab208670, 1:1000) in the presence of normal goat serum for 2 h at room temperature. Following quenching with 0.3% hydrogen peroxide, the slides were washed with 1 × TBS and subsequently incubated with a biotinylated secondary antibody. After incubating with an ABC reagent, the reaction was visualized by developing the slides with a DAB substrate solution.

### 4.7. Acute Toxicity Study

An acute toxicity study of the test compounds was performed using a variation of the mouse model of VVC as shown in Supplementary Materials, Figure S7. To this end, animals were estrogenized in a similar manner, but not inoculated with yeast on day 0. They were treated with PBS, vehicle, CHB4 or CHB6 (12.5 mg/kg) on days 3, 4 and 5 and euthanized on day 6. Tissues were then collected and fixed for H&E staining.

### 4.8. In Vitro Skin Irritation Test

The in vitro skin irritation test was performed using MatTek Corporation’s Reconstructed Human Epidermal Model EpiDerm™ following the manufacturer’s instructions, which fulfills the criteria set forth in the Organization for Economic Co-operation and Development [27].

Briefly, we applied either 30 µL of CHB4 or CHB6, PBS, 0.1% DMSO or 30 µL of 1% Triton-X100 (positive control for irritation). All plates were then incubated in a humidified environment for an additional 35 ± 1 min at 37 ± 1 °C, 5 ± 1% CO_2_, and 90% ± 10% relative humidity (RH). Once the 1 h incubation period was completed for the dosed tissues, all plates were removed from the incubator and placed in a sterile hood. The tissues were then rinsed 15 times with sterile PBS. The blotted tissue inserts were then incubated for 42 ± 2 h after which time the cell viability was determined using the MTT assay as described [27].

### 4.9. Statistical Analysis

The results of in vivo studies are presented as mean ± the standard error of the mean (S.E.M.) for all the samples. The results of in vitro studies are presented as mean ± the standard error of the mean (S.E.M.) of at least three experiments carried out in triplicates. The data were analyzed by one-way ANOVA followed by Dunnett’s multiple comparison test using GraphPad Prism 8.0. Statistical significance was considered at *p* < 0.05.

## 5. Conclusions

Two *N*-terpenyl organoselenium compounds, CHB4 and CHB6, have demonstrated significant effectiveness as potential antifungal candidates. These compounds inhibited the growth of both FLU-sensitive (S1) and FLU-resistant (S2) strains of *C. albicans*. In addition, CHB4 effectively inhibited the activity of Pma1p, a vital enzyme in *C. albicans*.

In vivo findings using a mouse model demonstrated the possible therapeutic potential of CHB4 and CHB6 in treating VVC via the intravaginal route of administration. CHB4 and CHB6 effectively reduced vaginal yeast infection and alleviated inflammation and damage to the vaginal mucosa, respectively. The reduced expression of MPO in vaginal tissues from mice treated with CHB4 or CHB6 indicated a significant decrease in the inflammatory response, resulting in a lower presence of PMNs while not causing irritation to the mouse vaginal tract or in a reconstructed human epidermis model.

Overall, our study presents CHB4 and CHB6 as promising drug candidates for the treatment of VVC.

## 6. Patents

No patents have been submitted.

## Figures and Tables

**Table 1:** Molecular properties of test compounds and EB ^a^.

Compounds	Molecular Weight (g/mol)	Water Solubility (µg/mL)
CHB1	334.31	4.78
CHB2	334.31	4.78
CHB3	334.31	6.07
CHB4	334.31	4.78
CHB5	334.31	6.30
CHB6	336.33	2.75
EB	274.18	33.00

^a^ Prediction Swiss ADME platforms (http://www.swissadme.ch/, accessed on 1 October 2023).

**Table 2:** MIC from turbidity assay in S1 and S2 strains of *C. albicans*.

Compounds	*C. albicans* S1 (µM)		*C. albicans* S2 (µM)	
	24 h	48 h	24 h	48 h
CHB1	12.5	12.5	3.1	3.1
CHB2	12.5	12.5	12.5	12.5
CHB3	25.0	25.0	50.0	25.0
CHB4	12.5	12.5	3.1	3.1
CHB5	25.0	12.5	12.5	25.0
CHB6	3.1	3.1	3.1	3.1
EB	25.0	25.0	25.0	25.0
FLU	25.0	25.0	>100	>100

**Table 3:** MIC from colorimetric assay in S1 and S2 strains of *C. albicans*.

Compounds	*C. albicans* S1 (µM)		*C. albicans* S2 (µM)	
	24 h	48 h	24 h	48 h
CHB1	12.5	6.3	12.5	12.5
CHB2	12.5	12.5	6.3	3.1
CHB3	12.5	25.0	25.0	25.0
CHB4	6.3	12.5	3.1	3.1
CHB5	12.5	25.0	12.5	12.5
CHB6	3.1	3.1	6.3	6.3
EB	25.0	25.0	25.0	25.0
FLU	>100	>100	>100	>100

**Table 4:** Viability assay evaluated in vitro *.

Compounds	MTT Assay IC_50_(µM) KB-3-1 Cells	Turbidity Assay IC_50_ (µM)	
		*C. albicans* S1	*C. albicans* S2
CHB1	28.1 ± 1.5	7.3 ± 0.1	1.8 ± 0.1
CHB2	25.5 ± 1.9	7.6 ± 0.1	6.2 ± 0.1
CHB3	65.7 ± 4.4	18.1 ± 0.2	15.1 ± 0.2
CHB4	17.9 ± 3.1	7.7 ± 0.1	1.8 ± 0.1
CHB5	94.1 ± 4.7	7.5 ± 0.1	14.4 ± 0.5
CHB6	48.5 ± 5.9	1.8 ± 0.1	1.8 ± 0.1
EB	89.74 ± 3.42	17.06 ± 1.01	14.28 ± 0.46
FLU	>1000	6.01 ± 0.10	>100

* IC_50_ is defined as the drug concentration that inhibits microbial growth by 50% as compared to untreated cells.

**Table 5:** Medium acidification assay in *C. albicans* S1 ^a^.

Compound	IC_50MA_, µM
CHB1	>30
CHB2	>30
CHB3	>30
CHB4	13.8 ± 1.1
CHB5	>30
CHB6	>30
EB	12.5 + 1.1

^a^ Values are reported as mean of three experiments ± standard error of the mean (SEM). The highest test concentration for each compound was 30 µM; therefore, if the compound failed to reach an IC_50MA_ value, it is listed here as >30 µM.

**Table 6:** Growth inhibition of *C. albicans* S1 strain in the VVC mouse model.

Groups	Log CFU/100 μL	Remaining *C. albicans* S1 Compared to Control (100%)
6th day infected (control)	5.628	100.000
Miconazole (MICO)	4.470	6.950
Vehicle	5.630	99.541
CHB4	4.448	6.607
CHB6	3.655	1.064
Naive	-	-

## Data Availability

Data is contained within the article and Supplementary Materials.

## Supplementary Materials

The following supporting information can be downloaded at: https://www.mdpi.com/article/10.3390/molecules28217377/s1, Figure S1: HPLC analysis of the test compounds CHB1-CHB3; Figure S2. HPLC analysis of the test compounds CHB4-CHB6; Figure S3. Representative image of colorimetric assay in *C. albicans* S1 after 48 h incubation; Figure S4. Effects of test compounds on Na^+^, K^+^-ATPase activity; Figure S5. Experimental design for VVC; Figure S6. Acute toxicity analysis was performed as indicated in Figure S5 without the inoculation of *C. albicans* S1 on day 0.
