# Supplementary material for: Selected N-Terpenyl Organoselenium Compounds Possess Antimycotic Activity In Vitro and in a Mouse Model of Vulvovaginal Candidiasis
Source: Molecules. 2023 Oct 31;28(21):7377. doi: 10.3390/molecules28217377 (PMC10647704; doi:10.3390/molecules28217377)
Supplement: Supplementary file 1 [file molecules-28-07377-s001.zip › Supplement-CHB paper_NFedit_BBedit_OCTOBER 19_2023.pdf]

<u>Supplementary Materials</u>:

Selected *N*-terpenyl Organoselenium Compounds Possess Antimycotic Activity *In Vitro* and in a Mouse Model of Vulvovaginal Candidiasis

Xiuyi Liang[1], Agata J. Pacuła-Miszewska[2], Magdalena Obieziurska-Fabisiak[2], Richa Vartak[1], Ganming Mao[1], Ketankumar Patel[1], Natalya U. Fedosova[3], Jacek Ścianowski[2] and Blase Billack[1,*]

SUPPLEMENTARY DATA

1. HPLC analysis

Chromatographic separation of CHB 1-6 was performed using Waters alliance® HPLC equipped with 2998 Photodiode Array (PDA) detector and Hypersil® ODS column (250 mm × 4.6 mm, 5 µm). An optimum ratio of 60:40 Acetonitrile (ACN): HPLC grade water was used as the mobile phase. Injection volume and flow rate were set to 10 µL and 1 ml/min respectively. Column temperature was maintained at 25°C. Samples were injected and analyzed using autosampler and output signal was detected using Empower 3 software at 265 nm.

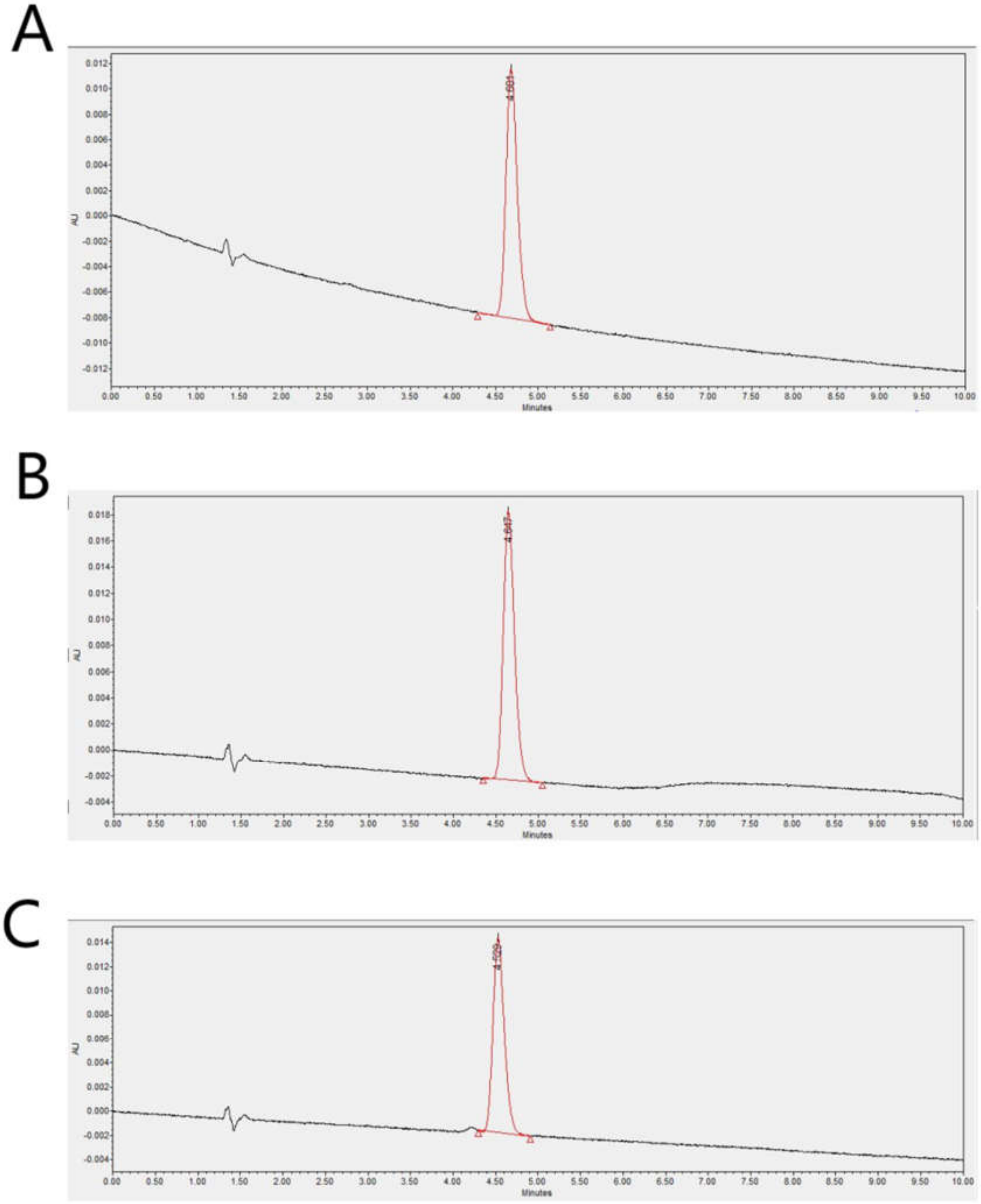

Figure S1. HPLC analysis of the test compounds. (A) CHB1 (retention time = 4.7 ± 0.1 min); (B) CHB2 (retention time = 4.6 ± 0.1 min); (C) CHB3 (retention time = 4.5 ± 0.1 min).

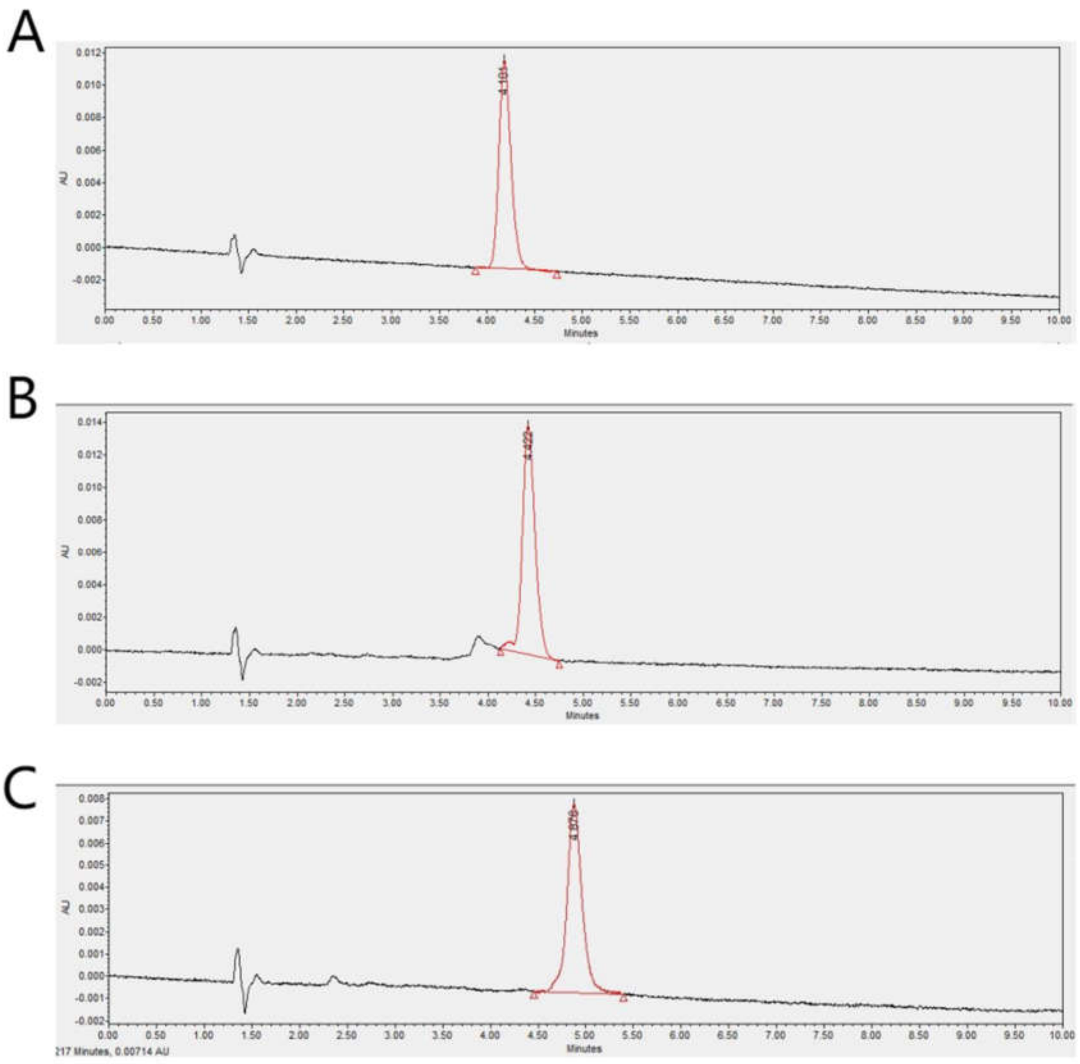

Figure S2. HPLC analysis of the test compounds. (A) CHB4 (retention time = 4.1 ± 0.1 min); (B) CHB5 (retention time = 4.4 ± 0.1 min); (C) CHB6 (retention time = 4.9 ± 0.1 min).

2. Representative image of colorimetric assay

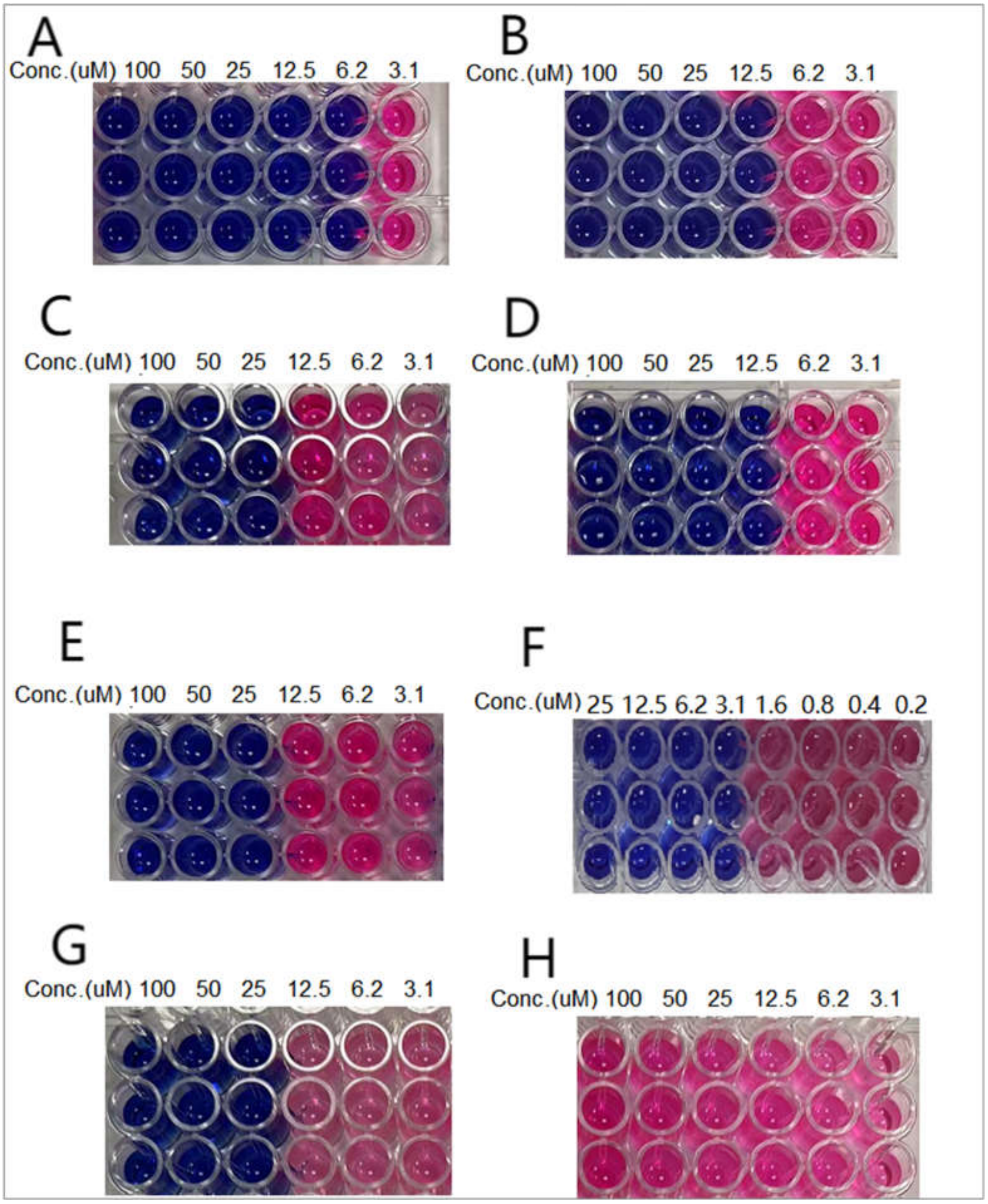

Figure S3. Representative image of colorimetric assay in *C.albicans* S1 after 48 h incubation. Living yeasts reduce the resazurin dye to the fluorescent product resorufin (pink) while the wells with dead yeast cannot reduce the dye and remain blue in color. (A) CHB1; (B) CHB2; (C) CHB3; (D) CHB4; (E)CHB5; (F) CHB6; (G) EB; (H) FLU.

3. Measuring Na^+, K^+-ATPase activity

Na^+, K^+-ATPase was purified from pig kidney outer medulla. The interactions with the inhibitors were estimated from the decrease of the ouabain-sensitive hydrolytic activity. The assays were carried out at 37 °C in a medium consisting of 130 mM NaCl, 20 mM KCl, 4 mM $MgCl_2$ and 20 mM histidine (pH 7.4). The enzyme was pre-incubated with the compounds for 1 h at 37 °C prior to addition of 3 mM ATP, whereupon hydrolysis of ATP was allowed to proceed for 2 min. In the time-dependent batch experiments, i.e. binding of the compounds or re-activation of the ATPase by 5 mM GSH, the enzyme samples were taken after different periods of time and investigated for the residual activity by measuring ATP hydrolysis for the same 2 min. Specific Na^+, K^+-ATPase activity was calculated as difference in Pi release in the absence and presence of 1 mM ouabain. The reaction is irreversible as illustrated by Figure S4, panel C where the enzyme activity is not restored by addition of 5 mM GSH after complete inactivation is reached.

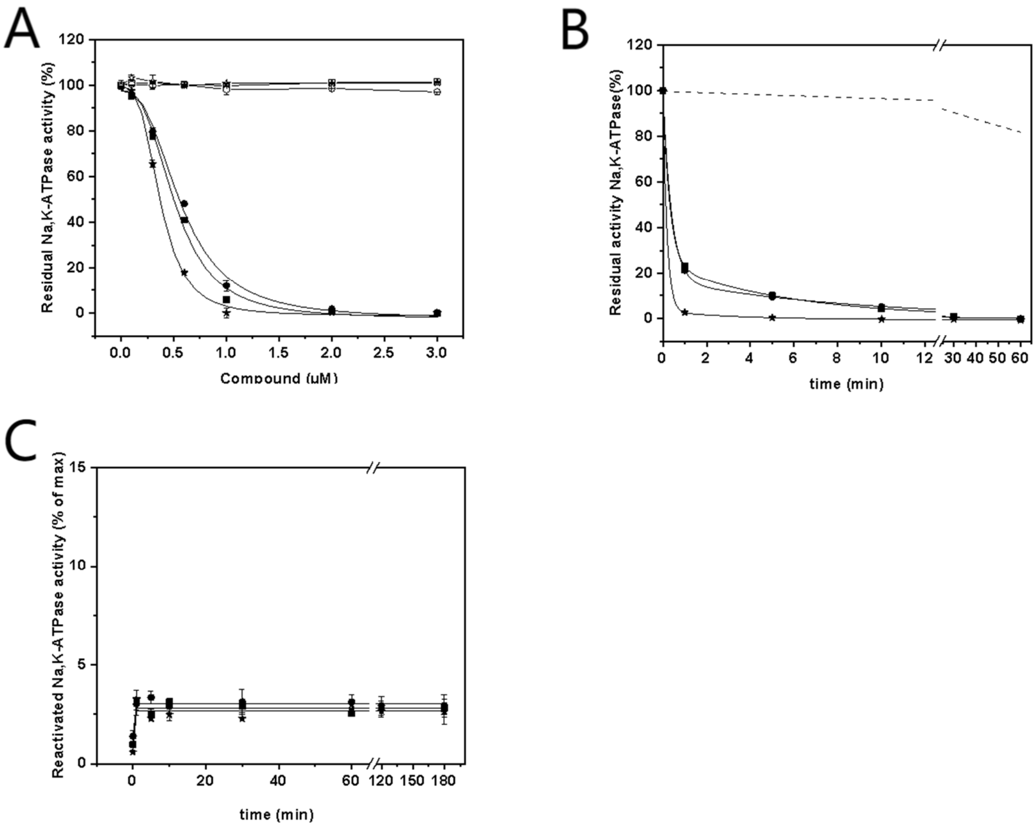

Figure S4. Effects of test compounds on Na^+,K^+-ATPase activity. (A) Inactivation of the Na^+,K^+-ATPase by ebselen and its derivatives in the absence (lower curves) and presence (flat lines) of 5 mM GSH. (B) Time course of inactivation of the Na^+,K^+-ATPase by 3 μM ebselen or its analogs. Non-specific inhibition by DMSO is shown as a broken line. (C) Time course of re-activation of the Na^+,K^+-ATPase by addition of 5 mM GSH. CHB4 (squares), CHB6 (circles), EB (stars). The data are averages of three independent experiments with the error whiskers representing SD.

4. Study design for intervention treatment of murine VVC

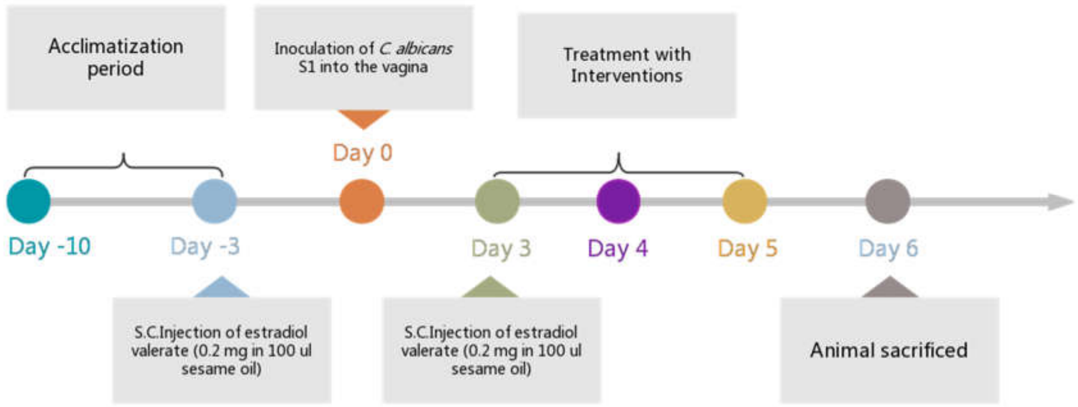

Figure S5. Animals were acclimatized from Day -10 to Day -3 and were administered with subcutaneous injection of estradiol valerate except for the naive group on day -3. On Day 0, animals were intravaginally inoculated with *C. albicans* S1 strain (5.5 x 10^5 CFU/20 µL); On Day 3, mice received a second subcutaneous injection of estradiol valerate except for the naive group and mice from the Day 3 group which were euthanized. Day 3 also marks the start of the intervention treatment to the respective treatment groups and was continued until Day 5. On Day 6, the remaining mice were euthanized, the vaginal lavage fluid was collected, and the vaginal tissues were excised for further studies.

5. Acute toxicity study

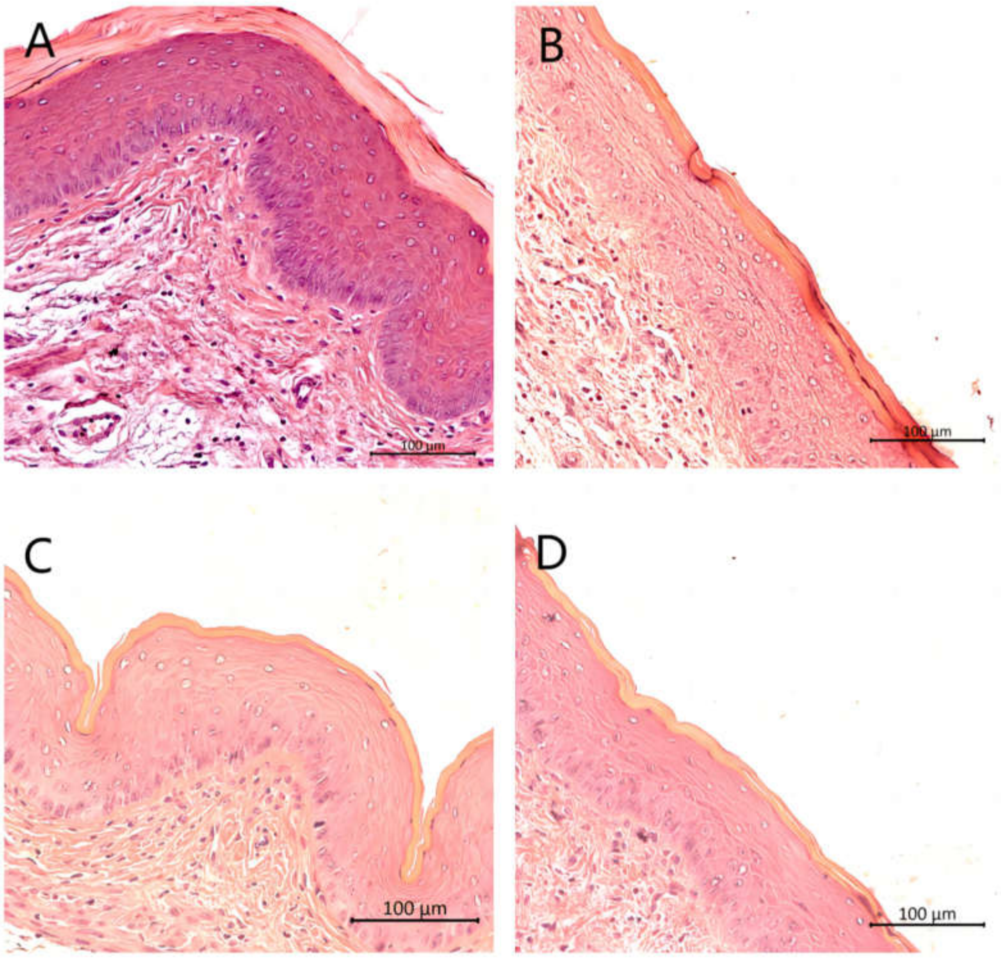

Figure S6. Acute toxicity analysis was performed as indicated in Figure S1 without the inoculation of *C. albicans* S1 on day 0. Each section of paraffin-embedded tissues was stained with H & E and then observed using light microscope. (A) PBS treatment; (B) Vehicle treatment; (C) CHB4 (12.5 mg/kg) treatment; (D) CHB6 (12.5 mg/kg) treatment. Magnification: 200×; Scale bars: 100 µm.
